# Oral Submucous Fibrosis: A Review on Etiopathogenesis, Diagnosis, and Therapy

**DOI:** 10.3390/ijms20122940

**Published:** 2019-06-16

**Authors:** Yin-Hwa Shih, Tong-Hong Wang, Tzong-Ming Shieh, Yu-Hsin Tseng

**Affiliations:** 1 Department of Healthcare Administration, Asia University, Taichung 41354, Taiwan; evashih@asia.edu.tw; 2 Tissue Bank, Chang Gung Memorial Hospital, Taoyuan 33305, Taiwan; cellww@adm.cgmh.org.tw; 3 Research Center for Industry of Human Ecology, Chang Gung University of Science and Technology, Taoyuan 33303, Taiwan; 4 Graduate Institute of Health Industry Technology, Chang Gung University of Science and Technology, Taoyuan 33303, Taiwan; 5 Liver Research Center, Chang Gung Memorial Hospital, Taoyuan 33305, Taiwan; 6 Department of Dental Hygiene, College of Health Care, China Medical University, Taichung 40402, Taiwan; 7 Department of Pediatrics, Kaohsiung Medical University Hospital, Kaohsiung Medical University, Kaohsiung 80708, Taiwan

**Keywords:** collagen deposition, diagnostic biomarkers, oral submucous fibrosis (OSF), precancerous disorder, therapeutic interventions, underlying mechanisms

## Abstract

Oral submucous fibrosis (OSF) is characterized by abnormal collagen deposition. It is a precancerous disorder and transforms into a malignant tumor in 1.5–15% of all cases. Symptoms include submucous fibrosis, ulceration, xerostomia, a burning sensation, and restricted mouth opening. All of these greatly interfere with patient quality of life. The present review introduces OSF from a molecular perspective and summarizes what is known about its underlying mechanisms, diagnostic biomarkers, and therapeutic interventions. In addition to the aggressive treatment of OSF, its prevention is also important. Future research should, therefore, focus on improving the oral health literacy of the patients susceptible to OSF.

## 1. Introduction

Oral submucous fibrosis (OSF) is a chronic disease that produces scars, tissue fibrosis, and precancerous lesions. It frequently occurs in the buccal mucosa [1,2]. Pathological characteristics include chronic inflammation, excessive collagen deposition in the connective tissues below the oral mucosal epithelium, local inflammation in the lamina propria or deep connective tissues, and degenerative changes in the muscles. OSF patients experience a severe burning sensation in the mouth after ingesting spicy foods. Other symptoms of OSF include dry mouth, pain, taste disorders, restricted tongue mobility, trismus, dysphagia, and altered tone. This disease contributes significantly to mortality because of its high malignant transformation rate (1.5–15%) [3]. The incidence of OSF differs with ethnicity and region and is closely associated with diet, habits, and culture [4,5,6]. India has the greatest number of OSF patients worldwide but the disease also occurs in Taiwan and other Asian countries [7,8]. There are also numerous OSF patients in South Africa as this country has many Indian immigrants. According to World Health Organization (WHO) statistics, there are >5 million OSF patients globally [9,10]. In India, OSF occurs more often in women than men but the opposite is true for other regions. The patient age range is 20–40 y.

Causative factors of OSF include autoimmunity, vitamin B, C, and iron deficiencies, chewing betel nut, consumption of spicy foods, human papilloma virus (HPV) infection, and genetic mutations [11,12,13,14,15]. Epidemiological studies have shown that chewing betel nut is one of the most significant risk factors for OSF [16]. Among OSF patients in China, 62.3% have the habit of chewing betel nuts [17]. Certain studies also reported that habits such as chewing and smoking tobacco and drinking alcohol increase the risk of OSF [12,18]. A study in Taiwan indicated that a high proportion of betel quid chewers are also tobacco smokers (86%) or alcohol drinkers (74%) [19]. Chewing betel nut and tobacco together substantially increases the incidence of OSF [20]. Other studies confirmed that drinking alcohol and chewing betel nut have an additive effect on OSF induction [19,21].

OSF is widely recognized as a precursor to oral precancer. Previous studies found that OSF patients in China have a 1.19% chance of developing oral cancer. In India, ~7.6% of all OSF patients develop oral cancer [8,22]. Previous studies proved that the duration of OSF and the extent to which its symptoms worsen are directly correlated with oral cancer progression. OSF generally progresses to oral cancer 3–16 y after the initial OSF diagnosis [23,24]. Unfortunately, there are no effective treatments for OSF available for clinical use. Here, the aim of this work is review the existing literature on the pathogenesis, molecular diagnosis, and clinical treatment of OSF in order to elucidate effective molecular prevention, diagnosis, and treatment strategies for it.

## 2. Pathological Symptoms and Molecular Mechanism

### 2.1. Pathological Symptoms

Chewing betel nut is the main cause of OSF [25,26,27,28]. The histopathology of OSF comprises various epithelial alterations, rete-peg shapes, and subepithelial deposition of dense bands of collagen fibers. At different OSF stages, epithelial alterations vary from atrophy with hypoplasia to hyperplasia and/or dysplasia [29,30]. A shift in epithelial compliance in response to increased connective tissue fibrosis favors the initiation of carcinomatous processes such as epithelial-mesenchymal transition (EMT) [31,32]. The most common initial symptoms of OSF are ulceration, xerostomia, a burning sensation, and limited ability to open the mouth [27,33]. These effects interfere with the daily life of the patient and may lead to complications. After tissue injury, myofibroblasts differentiate into contractile and secretory cells to close the wounds, produce components of the extracellular matrix (ECM) and secrete cytokines. However, excessive accumulation of ECM proteins such as collagen may result in pathological fibrosis [34,35].

### 2.2. Defective Collagen Homeostasis

**Figure 1:**
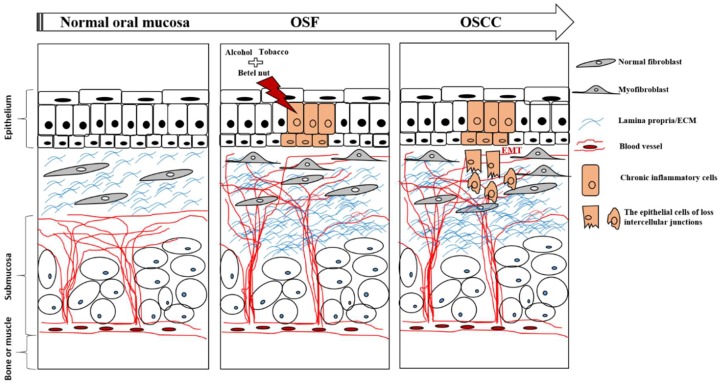
The molecular pathologic mechanism of oral submucous fibrosis (OSF).

Several studies confirmed that OSF is the result of collagen dysregulation, namely, increased biosynthesis and reduced clearance [12,25,36]. Betel nut contributes to these alterations in collagen metabolism. Betel nut contains alkaloids, flavonoids, and copper. All of these interfere with ECM homeostasis in oral tissue [12]. A high proportion of betel nut chewers also smoke tobacco and drink alcohol. Studies confirmed that tobacco smoking and alcohol consumption have an additive effect on OSF pathogenesis [19]. A schematic diagram of the molecular pathology mechanism of OSF is shown in Figure 1.

#### 2.2.1. Increased Collagen Synthesis

The four main alkaloids in betel nut are arecoline, arecaidine, guvacine, and guvacoline. These stimulate fibroblasts to produce collagen [12,16,37]. Both OSF- and normal cells produce ~85% type I collagen and ~15% type III collagen. In OSF cells, however, the ratio of the α1(I) to α2(I) chains of type I collagen is ~3:1 whereas in normal cells it is ~2:1 [38,39]. Moreover, the addition of slaked lime (calcium hydroxide) to betel nut in pan facilitates the hydrolysis of arecoline to arecaidine. The latter amplifies fibroblastic proliferation and increases collagen formation [35].

#### 2.2.2. Reduced Collagen Clearance

Collagen clearance is reduced by collagen stabilization, defective ECM dynamics, and inhibition of phagocytosis. Arecoline promotes the formation of cross-links between collagen peptide chains which renders the collagen resistant to degradation by collagenases [40,41]. The active constituents in betel nut induce substantial amounts of collagen synthesis in the oral mucosal cells. Cystatin C inhibits cysteine proteinases and might also stabilize collagen fibrils in OSF [42]. Arecoline upregulates cystatin C in buccal mucosal fibroblasts in a dose-dependent manner [12].

Fibroblast phagocytosis plays an important role in the regulation of ECM remodeling by collagen degradation [12]. However, fibroblasts from patients with OSF are markedly deficient in collagen phagocytosis. This defect may result in fibrosis [12,16]. It was demonstrated that collagen phagocytosis was inhibited in OSF fibroblasts treated with arecoline [12,35]. Flavonoids such as tannins and catechins are other important components of betel nut and work synergistically with alkaloids to induce OSF. Flavonoids stabilize collagen by inhibiting collagenase and stabilizing collagen fibrils [36]. Localized mucosal inflammation induced by betel nut recruits activated T cells and macrophages and increases cytokines and TGF-β. TGF-β significantly increases collagen production by activating the procollagen genes *COL1A2*, *COL3A1*, *COL6A1*, *COL6A3*, and *COL7A1*. It also increases procollagen proteinase and upregulates lysyl oxidase (LOX) which cross-links collagen fibers [12,35]. TGF-β also impedes collagen degradation by activating the tissue inhibitor of the matrix metalloproteinase (TIMP) gene and the plasminogen activator inhibitor (PAI) [25,36]. Decreased levels of gelatinolytic proteinases such as MMP (matrix metalloproteinase)-2 and MMP-9 secreted by fibroblasts and increased levels of TIMP-1 contribute to the loss of ECM equilibrium in OSF [35,43]. Arecoline significantly elevates TIMP-1 protein and mRNA expression in buccal mucosal fibroblasts [43]. Copper promotes LOX activity in OSF [44,45]. All of these significantly increase collagen production and maintenance [36,46].

### 2.3. Inflammatory Cytokines and Growth Factors

During betel nut chewing, the thick fibers injure the oral mucosa which causes inflammation of epidermal cells and activates macrophages to secrete cytokines. Transforming growth factor-β (TGF-β) is a major cytokine involved in OSF progression. It regulates the expression of α-SMA and type 1 collagen in myofibroblasts [47,48,49]. Studies have shown arecoline induced connective tissue growth factor (CTGF) biosynthesis via reactive oxygen species (ROS) and the NF-κB, JNK, and p38 MAPK pathways in buccal mucosal fibroblasts [35,48]. CTGF overexpression in individuals with the betel nut chewing habit may enhance fibrotic activity and pathogenesis in OSF [12,35]. Arecoline also upregulates other proinflammatory and profibrotic cytokines such as IL-1, IL-6, IL-8, TNF-α, PDGF, b-FGF, and KGF-1. It downregulates IFN-γ which, in turn, promotes collagen synthesis [12,50]. Changes in cytokines and growth factors cause fibroblast proliferation and collagen synthesis near the site of injury, thereby resulting in fibrosis [51,52,53]. In addition to the classic targets of fibrosis—TGF-β, IL-6 and more—a large amount of evidence from across different tissues, such as heart, lung, skin, liver, colon, and kidney, indicated that IL-17 and its downstream pathways are closely related to the initiation and propagation of fibrosis [54]. The role of IL-17 in the progression of OSF has not yet been explored, and this subject is worthy to investigate in the future.

### 2.4. Malignant Transformation

OSF constitutes a failure in the wound healing process following chronic persistent injury to the oral mucosa [55]. Paymaster first identified the malignant potential of OSF in 1956 but its mechanisms have not yet been elucidated [28]. The high mortality rate associated with oral squamous cell carcinoma (OSCC) is the result of late diagnosis of the malignant potentiality of its associated precancers [29,56]. Malignant transformation in the OSF background is complex. It involves numerous pathways and molecules associated with hypoxia, the cell cycle, angiogenesis, and epithelial mesenchymal transition [57].

#### 2.4.1. Hypoxia

It was proposed that hypoxia is an important microenvironmental factor in OSF associated with betel quid chewing and its malignant transformation [57,58,59,60]. Hypoxia-inducible factor-1α (HIF-1α) is a key regulator of cellular responses to hypoxia and is strongly upregulated in various fibrotic diseases including OSF [61]. HIF-1α also participates in the upregulation of various growth factors associated with fibrogenesis such as vascular endothelial growth factor (VEGF), TGF-β, fibroblast growth factor (FGF), platelet-derived growth factor (PDGF), and epidermal growth factor receptor (EGFR). Hyperbaric oxygen treatment (HBO) increases oxygen tension and delivery to oxygen-deficient tissues and may serve as a supplementary therapy for fibrogenesis involving hypoxia [57,62].

#### 2.4.2. Cell Cycle

The proliferating cell nuclear antigen (PCNA) index is positively correlated with the malignant transformation potential. The PCNA index was higher in OSF epithelium than normal oral mucosa. There was a significant difference in the expression levels between the dysplastic OSF group and the nondysplastic group [57]. Cyclin B1, p34 (cdc2), and *p*-survivin play key roles in carcinogenesis by influencing mitosis in the G2/M phase [63,64,65]. All of these molecules were upregulated in OSF relative to normal mucosa and there was a significant difference between OSF and OSCC in terms of their expression levels. P63 is consistent with p53 and regulates cell proliferation and differentiation. It serves as a surrogate marker of malignant transformation [66]. Nuclear positivity to p63 consistently increased with OSF progression to OSCC. Thus, p63 is a quantitative biomarker of the malignant potential and progression of OSF [67]. HPV infection is another factor leading to p53 inactivation. The E6 and E7 oncoproteins of high-risk HPV inactivate the p53 and retinoblastoma tumor suppressor proteins, resulting in loss of control of the cell cycle [68]. The prevalence of any HPV type in precancerous lesions (including OSF) was found to be higher than in the healthy control samples. However, only high-risk HPV types (types 16, 18, 31, 33, 35, 39, 45, 52) have a higher prevalence in OSCC lesions. This shows that high-risk HPV types are prevalent in OSCC and may play a role in its progression, while low-risk types are associated with oral pre-cancerous lesions [69].

#### 2.4.3. Angiogenesis

Multiple angiogenesis-related molecules such as inducible nitric oxide synthase (iNOS), b-FGF, TGF-β, PDGF, and HIF-1α are expressed in OSF and maintain the vascularity of the underlying connective tissue [57]. An increase in vasculature is an adaptive response of the mucosa to hypoxia induced by progressive fibrosis. However, once the malignant transformation has already occurred, it will promote tumor proliferation [70]. Arecoline toxicity may reduce vascularity in OSF. Conventional histology and morphological analysis demonstrated that mucosal vascularity declines with advanced fibrosis and increases in the juxtaepithelial area when dysplasia appears in the epithelium [71].

#### 2.4.4. Epithelial Mesenchymal Transition

EMT is a complex process involving the loss of cell-cell attachment, polarity, and specific epithelial markers, cytoskeletal remodeling, and the establishment of a mesenchymal phenotype [72]. EMT may be involved in the malignant transformation of OSF. EMT activators participate in betel nut associated OSF [27,55,73,74]. EMT-inducing transcription factors such as snail, slug, and twist are involved in the pathogenesis of betel nut related OSF. They initiate and facilitate the acquisition of a mesenchymal phenotype by repressing E-cadherin [27,73,75]. Zinc finger E-box binding homeobox 1 (ZEB1), another EMT-inducing transcription factor, is also implicated in betel nut associated OSF pathogenesis because it activates the α-SMA promoter [74].

## 3. OSF Diagnosis

### 3.1. Differential Diagnosis

Clinical, functional, and histological staging/classification of OSF have been documented. Certain staging systems are used by doctors for clinical OSF diagnosis or treatment [76]. In clinical staging/classification, early OSF presents with stomatitis and vesiculation, moderate OSF has a marble-like appearance and palpable fibrous bands, and severe OSF is manifested by leukoplakia and erythroplakia. In functional OSF classification, stages I–V range from a maximum interincisal mouth opening of >35 mm to <5 mm. As OSF may transform to OSCC, solid biopsies are necessary to assist with clinical diagnoses and therapeutic planning. In histological staging/classification, the number and distribution of fibroblasts, collagen fibers, inflammatory cells, and blood vessels are used to determine whether OSF is at an early, intermediate, or advanced stage. Moreover, biomarkers such as proteins, mRNAs, and non-coding RNAs are applied towards OSF staging and classification. In recent years, liquid biopsies of sera and saliva have been used to extend the functionality of the measuring instruments. Bioinformatic analyses can also be implemented in real time, reduce invasive and/or noninvasive diagnostic techniques, and replace surgical solid biopsies in OSF diagnosis.

### 3.2. Solid Biopsy

Tissue staining is the most common method of obtaining histological images from solid biopsies. Biomarkers are detected by methylated PCR, real-time PCR, western blotting, and staining techniques. These are used to identify promoter methylation, gene and protein expression levels, and marker locations in the tissues.

#### 3.2.1. Hematoxylin and Eosin (H&E) Stain and Specific Stains

H&E staining is often used as a control for immunohistochemical (IHC) staining as it indicates whether tissue processing has been performed correctly and reveals any artifacts. It clearly elucidates basic tissue morphology by staining the nuclei and cytoplasm purple and pink, respectively. Pathologists make diagnoses based on H&E staining as well as other specific stains and IHC detection in particular cases. Epithelial alterations, rete-peg shapes, subepithelial depositions of dense bands of collagen fibers, and inflammatory cells are regarded by pathologists as OSF markers. The relative efficacies of Mallory’s, Masson’s, and Van-Gieson stains were compared against H&E staining on 30 OSF tissues. Mallory’s stains most effectively highlighted variations in the thick keratin layer of the stratified squamous epithelium, subepithelial edema, subepithelial hyalinization, fibrillar and homogeneous collagen, and areas of degenerating skeletal muscle bundles and hyalinization. However, it barely revealed constricted blood vessels [77].

#### 3.2.2. Coding Gene and Protein Biomarkers in OSF Tissues

Several pathways and molecules associated with hypoxia, the cell cycle, angiogenesis, and EMT are involved in OSF pathology. Most OSF cases presented with positive PCNA expression in the basal and suprabasal layers. In 77% of the cases, there was also positive PCNA expression in the superficial layer [78]. Proteomic two-dimensional electrophoresis (2-DE) identified cyclophilin A (CYPA) as a biomarker and gene intervention target of OSF [79]. CYPA participates in carcinogenesis. It may promote cell proliferation and inhibit apoptosis by caspase deactivation. The latter is a therapeutic target for OSF [80]. Nuclear receptor coactivator 7 (NCOA7) was selected by matrix-assisted laser desorption ionization imaging mass spectrometry (MALDI-IMS) analysis and confirmed by cell lines, animal models, and 32 pairs of OSCC tissues and their corresponding adjacent noncancerous OSF tissues. NCOA7-associated proteins regulated the cell cycle and cell proliferation. These are potential biomarkers for the early diagnosis of OSF malignant transformation [81].

Arecoline induces HIF-1α protein expression in a dose-dependent manner. HIF-1α expression was significantly upregulated in the fibroblasts, epithelial cells, and inflammatory cells of betel quid chewers. Activated HIF-1α stimulates PAI-1 expression and induces extracellular matrix accumulation leading to OSF [58]. CD105 is a TGF-β signaling receptor and plays important roles in angiogenesis and fibrogenesis. It is essential for endothelial cell proliferation and promotes angiogenesis. CD105 was a more specific biomarker than CD34 in the determination of OSF neoangiogenesis [82]. A combination of MALDI-MS, one-dimensional sodium dodecyl sulfate polyacrylamide gel electrophoresis (1D SDS-PAGE), and nano liquid chromatography (nanoLC) was used to identify α-enolase overexpression in biopsies of oral OSF with dysplasia relative to nondysplastic OSF and normal oral mucosa. The α-enolase promotes cell proliferation by regulating PI3K/AKT signaling, inducing tumorigenesis by activating plasminogen, and increasing the Warburg effect. High α-enolase expression in OSF tissues is detected by western blotting, IHC, and RT-qPCR [83].

Ki67 and cyclin D1 evaluate cell proliferation while p16 and p53 are tumor-suppressor genes. β-catenin and c-Jun are associated with transcriptional activity. The hepatocyte growth factor receptor c-Met and the insulin-like growth factor II mRNA-binding protein 3 (IMP3) are linked with tumor invasion. In OSF, Ki67, cyclin D1, c-Met, and IMP3 are upregulated but β-catenin is downregulated. Ki67 upregulation combined with p16 downregulation significantly differs between transforming and nontransforming OSF [84]. The secreted Wnt antagonist Wnt inhibitory factor-1 (WIF1) inhibits Wnt/β-catenin signaling by directly binding to Wnt proteins. WIF1 promoter methylation may account for the β-catenin activation characteristic of OSF carcinogenesis [85]. In contrast, promoter methylation suppresses the secretion of the frizzled-related proteins 1 (SFRP1) and SFRP5 and are coupled with cytoplasmic/nuclear β-catenin accumulation in OSF carcinogenesis [86]. β-catenin expression levels in the normal, hyperplastic, dysplastic, and OSCC stages of OSF merit further investigation.

#### 3.2.3. Non-Coding Gene Biomarkers in OSF Tissues

Certain microRNAs are stable in frozen or paraffin-embedded tissues and low copy numbers may nonetheless be analyzed by reverse-transcription qPCR. The miR-200b and miR-200c were downregulated in OSF specimens. Arecoline treatment reduced miR-200c expression in buccal mucosal fibroblasts. The miR-200c and miR-200b upregulated E-cadherin by targeting ZEB1 and ZEB2, respectively [87,88]. ZEB1 binds to the α-smooth muscle actin (α-SMA) promoter and induces α-SMA which is overexpressed in the myofibroblasts during fibrogenesis. LncRNA GAS5-AS1 was downregulated in OSF tissues. It repressed phosphorylated Smad2 and downregulated TGF-β/Smad signaling and α-SMA expression in myofibroblasts [89]. In contrast, LINC00974 had the opposite effects. LINC00974 was aberrantly upregulated in OSF tissues and myofibroblasts [90].

### 3.3. Liquid Biopsy

Current biochemical and biomolecular techniques are more stable and sensitive than their predecessors. Even low concentrations of free ions, circulating cells, proteins, nucleic acids, and enzymes may be detected in body fluids. Serum protein and globulin levels were significantly decreased in OSF relative to normal tissues [91]. Serum copper levels gradually increased as OSF transformed into OSCC along with the duration of the betel quid chewing habit [92]. Saliva samples are easily obtained from patients and have been used as diagnostic samples for the past decade. In recent years, OSF biomarkers have been identified in serum and saliva and the feasibility of their application in OSF diagnosis has improved as evidence and sample sizes have increased.

#### 3.3.1. Biomarkers in OSF Serum

The rates of sister chromatid exchange per lymphocyte in patients with OSF and pan chewers were significantly higher than those in healthy controls. ROS-induced DNA damage is responsible for genome instability [93]. The levels of the provitamin A carotenoid β-carotene decreased with OSF progression [94]. Erythrocyte superoxide dismutase (E-SOD) and glutathione peroxidase (GPx) levels were significantly lower in the OSF, oral leukoplakia, and oral cancer groups than the control [95]. Lactate dehydrogenase (LDH) catalyzes the oxidation of lactate to pyruvate and its levels are markedly elevated in several potentially malignant lesions/conditions and oral cancer [96]. Serum LDH levels were directly correlated with betel chewing frequency and mouth opening in OSF patients. On the other hand, no such associations were found for salivary LDH [97]. Serum LDH may be a better biological marker of OSF than salivary LDH. OSF patients presented with elevated DNA damage and lipid peroxidation levels compared with healthy controls. As malondialdehyde (MDA) is a lipid peroxidation marker, the evaluation of its levels by comet assay may help identify OSF patients with high malignant potential [98].

#### 3.3.2. Biomarkers in OSF Saliva

MDA and 8-hydroxy-2-deoxyguanosine (8-OHdG) are detectable in serum, urine, and saliva. Salivary 8-OHdG and MDA were higher in OSF patients but salivary vitamins C and E were lower in OSF patients than healthy normal controls. Multiple biomarkers may increase OSF diagnosis specificity and sensitivity and could include 8-OHdG, MDA, vitamin C, and vitamin E [99]. In another study, total salivary protein and lipid peroxides were higher but vitamins A, C, and E and salivary SOD and GPx were lower in OSF patients than the controls [100]. Thus, oxidative stress is correlated with OSF progress. Salivary LDH was significantly higher in the OSF group than the control [101]. S1007 was first isolated from squamous epithelial cells in psoriatic skin. The levels of salivary S100A7 were higher in OSF patients than the healthy normal group [102]. High S100A7 expression is observed in potentially malignant oral disorders and is associated with the risk of malignant transformation in oral dysplasia [103,104].

### 3.4. Instrumentation for OSF Diagnosis

Biopsies are usually performed to confirm clinical diagnoses. Certain patients with OSF refused incisional biopsy because of the low transforming rate of OSF. Therefore, autofluorescence spectroscopy, optical coherence tomography (OCT), and Fourier transform infrared spectroscopy (FTIR) were used to assist incisional biopsy in OSF diagnosis.

Autofluorescence spectroscopy exploits the fact that various diseased tissues have different and unique histomorphological characteristics. When tissues are excited to a suitable wavelength, intrinsic fluorophores rise to various fluorescence emission spectra. For example, the maximum emissions for tryptophan, collagen, and nicotinamide adenine dinucleotide (NADH) are measured at 340 nm, 380–400 nm, and 440–460 nm, respectively. The 330 nm excitation applied to OSF mucosa had a significantly higher 380 nm emission peak and a significantly lower 460 nm emission peak than those of normal oral mucosa [105]. There were significant statistical differences in the emission peaks between normal and OSF patients and between betel quid chewers and OSF patients [106]. After the OSF was treated, the mucosa presented with relatively lower intensity at ~385 nm and comparatively higher intensity at ~440 nm than untreated OSF mucosa [107].

In 1988, the time-domain OCT system was used for the first time on human teeth and oral mucosa. OCT uses low-coherence light to capture two- and three-dimensional images at micrometer resolution within optical scattering media. Epithelial thickness and the standard deviation (SD) of the A-mode scan intensity in the laminar propria layer are effective diagnostic markers for OSF [108].

### 3.5. Combining Instrumentation and Sera in OSF Diagnosis

FTIR generates infrared absorption and emission spectra of solids, liquids, and gases. Previously, as mentioned above, it is stated that serum protein, globulin, vitamin, copper, specific protein, enzyme, and nucleic acid levels significantly differed between OSF patients and healthy controls. Combining instrumentation with sera is more effective than either approach alone and may save time and reagents in OSF diagnosis. FTIR spectroscopy of sera from OSF patients could be useful in rapid and accurate preoperative screening/diagnosis [109].

At present, there are more biomarkers available for solid than liquid biopsies. They are associated with cell proliferation (PCNA, Ki67, cyclin D1, cyclophilin A, NCOA7, and α-enolase), angiogenesis (HIF-1α, PAI-1, and CD105), cell invasion (c-Met and IMP3), Wnt-dependent signaling (WIF1, SFRP1, SFRP5, and β-catenin), promoter methylation (WIF1, SFRP1, and SFRP5), microRNAs (miR-200b target ZEB2 and miR-200c target ZEB1), and lncRNAs (lncRNA GAS5-AS1 and lncRNA LINC00974 with activated and inhibited TGF-β/Smad signaling, respectively). Ki67 in combination with p16 and NCOA7 could detect high OSF transformation rates to malignant cells. Liquid biopsies are relatively less invasive and highly efficient. The protein and globulin levels in the sera of OSF patients are comparatively downregulated. The elevated copper levels seen in OSF patients may be a consequence of betel chewing. Genome instability markers are also used in OSF diagnosis. These include sister chromatid exchange in the lymphocytes of OSF patients. To this end, a comet assay for DNA damage detection is used. Anti-ROS enzymes (E-SOD, GPx), ROS products (MDA), and cellular metabolic enzyme (LDH) activities in sera differed between normal and OSF patients. Certain enzymes and ROS products such as GPx, SOD, LDH, MDA, and 8-OHdG were detectable in both sera and saliva. However, 8-OHdG was not detected in the sera of OSF patients. The levels of vitamins C and E were reduced in OSF saliva compared to those of normal controls. Upregulated and downregulated biomarkers are listed in Table 1. Techniques of molecular biology were used to screen for OSF biomarkers, and instrumentation such as autofluorescence spectroscopy, OCT, and FTIR was also adapted for OSF diagnosis.

## 4. Treatment

OSF is treated primarily with surgery and conservative methods including molecular approaches. This section is to discuss the conservative treatment of OSF using physical and drug therapies and natural compound remedies.

### 4.1. Physical Therapy

Hyperbaric oxygen therapy (HBOT) is used to treat decompression sickness, gas gangrene, and carbon monoxide poisoning. In HBOT, the patient is placed in a hyperbaric chamber in which the ambient oxygen pressure is higher than atmospheric pressure. HBOT was first applied in dentistry in 1988 to promote periodontal wound healing [110]. Recently, the application of HBOT in OSF was reported. HBOT enhances fibroblast apoptosis and inhibits fibroblast activity by reducing IL-1β and TNF-α production [111,112]. HBOT attenuates the production of proinflammatory cytokines such as IL-1, IL-6, and IL-10 [113]. HBOT enriches oxygenation of all tissues and hinders the production of reactive oxygen species such as E-SOD, GPx, catalase, paraoxonase, and heme-oxygenase-1 [114,115]. HBOT suppress fibroblast activity, has anti-inflammatory and antioxidant properties, thus resulting in the therapeutic effect of OSF [62].

### 4.2. Drug Therapy

The main objectives of drug therapy for OSF are anti-inflammation and degradation of the extracellular matrix. Corticosteroids comprise a class of steroid hormones produced in the vertebrate adrenal cortex. Many of them have been synthesized. The glucocorticoids and mineralocorticoid participate in numerous physiological and biochemical processes. Glucocorticoids block inflammation mediators and impede the inflammatory reaction [116]. They also block fibroblast proliferation and collagen deposition [117].

Dexamethasone, methylprednisolone, and betamethasone are synthetic drugs with glucocorticoid-like effects. Intralesional injection of synthetic corticosteroids significantly improves mouth opening [118,119] and alleviates the burning sensation [118,120] in OSF. Hyaluronidase and chymotrypsin are proteolytic enzymes that degrade extracellular matrices such as hyaluronan and collagen. They are usually co-administered with corticosteroids in OSF treatment [121,122]. Pentoxifylline is a xanthine derivative primarily used to mitigate muscle pain. It competitively and nonselectively inhibits phosphodiesterase, suppresses TNF-α production in lipopolysaccharide (LPS)-stimulated human monocytes [123], blocks leukotriene synthesis, and diminishes the inflammatory reaction. Pentoxifylline improved mouth opening and reduced the burning sensation in OSF [118]. It also facilitated swallowing and speech [124].

Colchicine has been used as early as 1500 BC to treat joint swelling. It was approved for medical use in 1961. It is extracted from the autumn crocus and decreases inflammation by inhibiting neutrophil activation and migration to the inflammation site and by suppressing IL-1 β activation [125]. The efficacy of colchicine in OSF treatment was first reported in 2013 [126]. Patients with OSF took 0.5 mg oral colchicine twice daily and received injections of 1500 IU hyaluronidase into each buccal mucosal lesion once weekly. By the second week, the burning sensation was alleviated, mouth opening increased, and histological parameters were reduced. The aforementioned dosages combined with 0.5 mL lignocaine hydrochloride once weekly improved mouth opening and reduced the burning sensation in patients with grade II OSF after 12 weeks [121].

### 4.3. Natural Compound Remedies

Natural compounds are pure chemical substances extracted from living organisms. Most natural compounds used to fight disease are extracted from herbs used in traditional Chinese medicine (TCM) and the foods we eat.

Compounds in TCM with potential efficacy against OSF include butylidenephthalide, glabridin, asiatic acid, tanshinone, and salvianolic acid B. Butylidenephthalide is extracted from *Angelica sinensis* or *Ligusticum chuanxiong* and has neuroprotective [127], vasorelaxant [128], and anticancer [129] effects and inhibits liver fibrosis and inflammation [130]. It has demonstrated therapeutic efficacy against OSF. In vitro tests revealed that butylidenephthalide downregulates α-SMA, fibronectin, and type 1 collagen A1 and reduces myofibroblast bioactivity [75]. Glabridin is derived from the root of *Glycyrrhiza glabra* (licorice). It is a type of isoflavonoid or natural phenolic compound with antioxidant and anti-inflammatory properties. It suppresses α-SMA, type I collagen, and TGF-β [131]. Asiatic acid is extracted from *Centella asiatica* which is also used in TCM. Asiatic acid ameliorated fibrosis of the liver [132] and lung [133] in vivo. It repressed TGF-β1, collagen 1 type 2, and collagen 3 type 1 in human buccal fibroblasts [134]. Tanshinone is obtained from *Salvia miltiorrhiza* which is the Chinese herbal Danshen. This material consists of dihydrotanshinone I, tanshinone I, and tanshinone IIA and has anti-inflammatory and antioxidant activity. Tanshinone epigenetically interacts with the p53 pathway which is downregulated in OSF [135]. Salvianolic acid B is also extracted from *Salvia miltiorrhiza*. In systemic sclerosis, it is antifibrotic and inhibits fibroblast proliferation and ECM gene transcription [136]. In a recent clinical trial, it was demonstrated that salvianolic acid B combined with corticosteroid improved mouth opening and reduced the burning sensation in OSF [137,138]. An in vitro study showed that salvianolic acid B inhibited collagen biosynthesis and increased collagen degradation [139].

Other natural compounds with potential anti-OSF efficacy include epigallocatechin-3-gallate (EGCG), aloe vera, curcumin, lycopene, and honey. EGCG is the most abundant catechin in tea. It is an antioxidant and suppresses cellular ROS [140]. In vitro studies showed that EGCG suppressed several fibrogenic genes such as early growth response-1, connective tissue growth factor, and transglutaminase-2 (TGM-2) [140,141,142,143]. Aloe vera is a succulent in the Liliaceae. It contains various minerals and vitamins and possesses anti-inflammatory activity. Aloe vera reduces the inflammasome formation in human macorphages [144]. Aloe vera is extensively applied in dentistry [145]. A meta-analysis disclosed that aloe vera alleviates the burning sensation of OSF during the first two months of treatment [146]. Curcumin is derived from the rhizomes of *Curcuma longa*. It is a natural phenolic compound commonly used as a dietary supplement and a food additive. Curcumin has anti-inflammatory, antioxidant, and anticancer properties. It suppresses the connective tissue growth factor TGF-β [147] and iNOS [148] and decreases cellular fibrogenic activity. Curcumin effectively ameliorates the burning sensation [149] and improves mouth opening [150] in OSF patients. Lycopene is a carotenoid found in tomatoes and watermelon. It reduces oxidative damage to lipids, proteins, and DNA. Ingestion of lycopene may mitigate oxidative stress in the entire body. A clinical trial indicated that oral lycopene intake improved mouth opening and alleviated the burning sensation in OSF patients [151,152]. Honey is a sweet and viscous food produced mainly by bees. No matter if in ancient times or in modern medicine, honey has been used to help wound healing with its anti-inflammatory, antioxidant, and anti-bacterial properties [153]. Honey inhibits the lipoxygenase [154], scavenges the free radicals [155], inhibits IL-1, IL-10 and COX-2 expression [154], and inhibits NF-κB signaling pathway [156]. Scientists apply honey against oral diseases such as halitosis, oral submucous fibrosis, chemotherapy-induced stomatitis, and radiotherapy-induced oral mucostitis [157]. Combining honey with turmeric significantly ameliorates the burning sensation of OSF patients [158]. Table 2 lists all known conservative OSF therapies and their molecular targets.

## 5. Conclusions

OSF is prevalent among Asians with a betel nut chewing habit. OSF disrupts collagen homeostasis by increasing the production and decreasing the clearance of collagen and inducing structural and compositional abnormalities. Aberrant oral submucous collagen deposition may be activated by inflammation, ROS production, and mutations. Clinical diagnosis of OSF is performed through functional and molecular pathology techniques. OSF symptoms include the loss of oral function manifested by restricted mouth opening, trismus, xerostomia, and dysphagia. Detection of OSF by molecular pathology methods focuses on biomarkers that induce abnormal collagen deposition and includes both invasive and noninvasive analyses. The invasive detection method identifies biomarkers in solid tissue and sera. The noninvasive method detects biomarkers in saliva and examines the mucosa using optical instruments. Molecular OSF treatment represses the biomarkers disrupting collagen homeostasis. In general, drug treatments for OSF are efficacious. Nevertheless, individuals susceptible to OSF and possibly to the further development of OSCC should abandon unhealthy lifestyle practices, such as betel nut chewing and tobacco smoking, and consume natural foods with anti-inflammatory and antioxidant properties.

## Figures and Tables

**Table 1:** Biomarkers in solid biopsy and liquid biopsy.

**Biomarker in Tissues**	**Note**	**Sample Size/References**
Up: PCNA	Cell proliferation	30 OSF, 10 OSCC [78]
Up: cyclophilin A	Cell proliferation	25 normal, 25 OSF [79,80]
Up: NCOA7	Cell proliferation, early diagnosis of OSF malignant transformation	24 OSF tissues without malignant transformation, 34 OSCC tissues arising from OSF [81]
Up: HIF-1α, PAI-1	Angiogenesis	6 normal, 25 OSF [58]
Up: CD105	Angiogenesis	15 normal, 30 OSF [82]
Up: α-enolase	Cell proliferation, tumorigenesis (increased Warburg effect)	13 normal, 19 OSF without dysplasia (OSFWT), 23 OSF with dysplasia (OSFWD), 28 OSCC [83].
Up: Ki67, cyclin D1, c-Met, IMP3Down: β-catenin	Cell proliferation (Ki67, cyclin D1), invasion (c-Met, IMP3), the combined biomarkers of Ki67 and p16 (tumor suppressor) showed significantly different expression between the transformation and non-transformation groups	6 normal, 36 OSF [84]
Down: WIF1	Wnt antagonist, inhibits Wnt/β-catenin signaling by directly binding to Wnt proteins.	15 normal tissue, 45 OSF, 55 OSCC [85]
Up: β-catenin Down: SFRP-1, SFRP-5	Wnt/β-catenin signaling	15 normal, 45 OSF, 55 OSCC [86]
Down: miR-200b, miR-200c Up: ZEB1, ZEB2	miR-200b targeting ZEB2, miR-200c targeting ZEB1	25 control, 25 OSF [87] 20 control, 20 OSF [88]
Down: LncRNA GAS5-AS1	LncRNA GAS5-AS1 bind to Smads and prevents them binding to SBE on TGF-β target gene promoter, thereby negatively regulates TGF-β/Smad signaling pathway	25 control, 25 OSF [89]
Up: LncRNA LINC00974	LncRNA LINC00974 activates TGF-β/Smad signaling	20 OSF tissues [48,90]
**Biomarker in Serum**	**Note**	**Sample Size/References**
Down: Serum protein, globulin	-	50 control, 50 nicotina stomatitis, 50 OSF, 50 leukoplakia, 50 malignancy [91]
Up: Copper	Serum copper levels increased gradually from precancer to cancer, as the duration of betel quid chewing habit increased.	30 control, 30 OSF, 30 OSCC [92]
Up: Sister chromatid exchange in lymphocytes	Genotoxic, genome instability,	10 male patients who had the habit of chewing pan for 5 or more years, 10 male patients with OSF who had panparag chewing habit and 10 age- and sex-matched controls without any chewing habit [93]
Down: β-carotene	β-carotene as the best-known provitamin A carotenoid	40 control, 40 OSF [94]
Down: E-SOD, GPx	Anti-ROS stress	25 control, 25 OSF, 25 leucoplakia, 25 OSCC [95]
Up: LDH	Cell metabolism	30 control, 30 OSF [96] 20 control, 20 OSF [97]
Up: MDA, comet assay	ROS product (MDA), DNA damage phenotype (comet assay)	30 control, 30 OSF [98]
**Biomarker in Saliva**	**Note**	**Sample Size/References**
Up: 8-OHdG, MDADown: Vitamin C, vitamin E	ROS product (8-OHdG, MDA), anti-ROS stress (vitamin C, vitamin E)	40 OSCC, 40 oral lichen planus lesions, 40 leukoplakia, 40 OSF, 40 control [99]
Down: GPx, SOD	Anti-ROS stress	63 control, 63 OSF [100]
Up: LDH	LDH main function is to catalyze the oxidation of lactate to pyruvate.	30 control, 30 OSF [96] 20 control, 20 OSF [97] 10 control, 25 OSF, 25 OSCC [101]
Up: S100A7	A small calcium-binding protein, has been associated with the development of psoriasis and carcinomas in different types of epithelia.	30 control, 30 OSF [102,103,104]
**Instrument**	**Note**	**Sample Size/References**
Auto-fluorescence spectroscopy, (320–330 nm excitation, collagen at 380–400 nm emission and NADH at 440–460 nm emission)	Up: Collagen Down: NADH	15 normal oral mucosa, 59 OSF mucosal [105]. 18 normal individuals without the habit of betel quid chewing, 18 betel quid chewers without OSF, 15 OSF [106]. 20 normal, 20 OSF [107].
OCT detect the epithelium thickness and the standard deviation of A-mode scan intensity in the laminar propria layer	Up: Epithelium thickness, laminar propria layer	44 OSF samples were obtained from 44 patients. Also, 44 healthy samples were obtained from 26 healthy volunteers. [108]
FTIR spectrometry	Protein contents in serum were different	30 control, 30 OSF [109]

**Table 2:** Summary of the conservative therapy of OSF and the molecular targets of each therapy.

**Physical Therapy**	**Molecular Targets**	**References**
Hyperbaric oxygen treatment (HBO)	Promote the apoptosis of fibroblast, and inhibit TNF-α, TGF-β, and the activation of collagen synthesis.	[62,111,112]
**Drug therapy**	**Molecular Targets**	**References**
Dexamethasone	Anti-inflammation (block the action of inflammatory mediators)	[119,159]
Methylprednisolone	Anti-inflammation (block the action of inflammatory mediators)	[119]
Betamethasone	Anti-inflammation (block the action of inflammatory mediators)	[120]
Hyaluronidase	Hydrolyze the hyaluronan	[121]
Chymotrypsin	Hydrolyze the collagen	[122]
Pentoxifylline	Anti-inflammation. Inhibits TNF-α and leukotriene synthesis	[123,160]
Colchicine	Anti-inflammation, neutralized cytokines (TGF-β, IL4, IL6) Increase collagenolytic activity	[126]
**Natural compounds remedies**	**Molecular Targets**	**References**
Butylidenephthalide	Decrease α-SMA and fibronectin and type 1 collagen A1 expression Inhibit myofibroblast activity (migration and contraction)	[75]
Glabridin	Decrease α-SMA, type I collagen, and TGF-β Inhibit myofibroblast activity (migration and contraction)	[131]
Asiatic acid	Inhibit TGF-β1, collagen 1 type 2, and collagen 3 type 1	[134]
Tanshinone	reactivate p53	[135]
Salvianolic acid B with Triamcinolone acetonide	Inhibit the transcription of procollagen gene COL1A1 and COL3A1 Decrease TIMP-1/-2 expression Inhibit the transcription and release of CTGF, TGF-β1, IL-6 and TNF-α Increase MMP-2/-9 activity	[137,138,139]
EGCG	Inhibit TGF-β1 to suppress early growth response-1 (Egr-1) Suppress the cellular ROS Inhibit the CTGF and TGM-2 expression	[140,141,142,143]
Aloe Vera	Anti-inflammation Reduce inflammasome formation	[161,162] [144]
Curcumin	Inhibit p53, TGF-β, and iNOS Reduce CTGF	[148,152]
Lycopene	Antioxidants	[151,152]
Honey	Anti-inflammation, anti-oxidation Inhibit lipoxygenase, IL-1, IL-10, COX-2 Scavenge free radicals Inhibit NF-κB signaling pathway	[153] [154] [155] [156]

## Abbreviations

OSF	oral submucous fibrosis
WHO	World Health Organization
HPV	human papilloma virus
EMT	epithelial-mesenchymal transition
ECM	extracellular matrix
TGF-β	transforming growth factor-β
LOX	lysyl oxidase
TIMP	tissue inhibitor of the matrix metalloproteinase
PAI	plasminogen activator inhibitor
MMP	matrix metalloproteinase
CTGF	connective tissue growth factor
ROS	reactive oxygen species
OSCC	oral squamous cell carcinoma
HIF-1α	hypoxia-inducible factor-1α
VEGF	vascular endothelial growth factor
FGF	fibroblast growth factor
PDGF	platelet-derived growth factor
EGFR	epidermal growth factor receptor
HBOT	hyperbaric oxygen therapy
PCNA	proliferating cell nuclear antigen
iNOS	inducible nitric oxide synthase
ZEB1	Zinc finger E-box binding homeobox 1
H&E	Hematoxylin and Eosin
IHC	immunohistochemical
2-DE	two-dimensional electrophoresis
CYPA	cyclophilin A
NCOA7	nuclear receptor coactivator 7
MALDI-IMS	matrix-assisted laser desorption ionization imaging mass spectrometry
nanoLC	nano liquid chromatography
IMP3	insulin-like growth factor II mRNA-binding protein 3
WIF1	Wnt inhibitory factor-1
SFRP1	secretion of the frizzled-related proteins 1
α-SMA	α-smooth muscle actin
E-SOD	erythrocyte superoxide dismutase
GPx	glutathione peroxidase
LDH	lactate dehydrogenase
MDA	malondialdehyde
8-OHdG	8-hydroxy-2-deoxyguanosine
OCT	optical coherence tomography
FTIR	fourier transform infrared spectroscopy
NADH	nicotinamide adenine dinucleotide
TCM	traditional Chinese medicine
TGM-2	transglutaminase-2
